# Supplementary material for: Resolving the full spectrum of human genome variation using Linked-Reads
Source: Genome Res. 2019 Apr;29(4):635–45. doi: 10.1101/gr.234443.118 (PMC6442396; doi:10.1101/gr.234443.118)
Supplement: Supplemental Material [file supp_gr.234443.118_Supplemental_longranger-2.2.zip › longranger-2.2.tar.bz2!/lr2.2-public/lib/stan/cmdstan/stan/doc/api/html/agrad_2rev_2matrix_2value__of_8hpp_source.html]

Stan: src/stan/agrad/rev/matrix/value\_of.hpp Source File


|  |  |
| --- | --- |
|  | Stan  2.5.0  probability, sampling & optimization |


- Main Page
- Related Pages
- Namespaces
- Classes
- Files

- File List
- File Members

All Classes Namespaces Files Functions Variables Typedefs Enumerations Enumerator Friends Macros Pages

- src
- stan
- agrad
- rev
- matrix

value\_of.hpp

Go to the documentation of this file.

1 #ifndef STAN\_\_AGRAD\_\_REV\_\_MATRIX\_\_VALUE\_OF\_HPP

2 #define STAN\_\_AGRAD\_\_REV\_\_MATRIX\_\_VALUE\_OF\_HPP

3

4 #include <stan/math/matrix/Eigen.hpp>

5 #include <stan/agrad/rev/var.hpp>

6

7 namespace stan {

8  namespace agrad {

15  template<int R,int C>

16  inline const Eigen::Matrix<double,R,C> &value\_of(const Eigen::Matrix<double,R,C> &M) {

17  return M;

18  }

19

26  template<int R,int C>

27  inline Eigen::Matrix<double,R,C> value\_of(const Eigen::Matrix<var,R,C> &M) {

28  int i,j;

29  Eigen::Matrix<double,R,C> Md(M.rows(),M.cols());

30  for (j = 0; j < M.cols(); j++)

31  for (i = 0; i < M.rows(); i++)

32  Md(i,j) = M(i,j).vi\_->val\_;

33  return Md;

34  }

35  }

36 }

37

38 #endif

stan::agrad::value\_of

T value\_of(const fvar< T > &v)

Return the value of the specified variable.

**Definition:** value\_of.hpp:16

var.hpp

Eigen.hpp


---

[ Stan Home Page ]

*© 2011–2014,
Stan Development Team.*
